# Commentary: Mastery is central: an examination of complex interrelationships between physical health, stress and adaptive cognition, and social connection with depression and anxiety symptoms

**DOI:** 10.3389/fpsyt.2024.1437227

**Published:** 2024-06-24

**Authors:** Yingying Zhou, Chang Xi

**Affiliations:** ^1^ School of Medicine, Hunan University of Chinese Medicine, Changsha, Hunan, China; ^2^ Department of Psychology, Hunan University of Chinese Medicine, Changsha, Hunan, China

**Keywords:** depression, anxiety, risk factors, resilience factors, sex differences

## 1 Introduction

The recent research article by Shin and Park (1) in Frontiers in Psychiatry investigated the complex pattern of interplay between depression and anxiety symptoms and pertinent physical, cognitive, and social factors and potential gender differences. They employed a network analysis to uncover various pathways linking risk and resilience factors with comorbid depression and anxiety symptoms, which would be particularly beneficial to provide prevention and interventions of depression and anxiety symptoms for middle-aged men and women. I wholeheartedly agree with this statement, and in this commentary, I advocate for the necessity of building the network model including physical, cognitive, and social factors and potential gender differences to analyze the interacting nature of different factors and depression and anxiety symptoms. It will yield significant benefits for the middle-aged with depression and anxiety symptoms in at least two crucial aspects: (1) based on sex differences in anxiety and depression network structure to tailor individualized interventions and improve psychopathology symptoms, and (2) by understanding central modifiable risk or resilience factors for better diagnosis and treatment, among which includes reducing perceptions of stress and promoting mastery.

## 2 Sex differences in anxiety and depression network structure

The findings of the sex differences in anxiety and depression network structure attach great importance to understanding the mechanisms of affective disorders symptoms and developing more targeted interventions.

Sex differences were prominent in mood and anxiety disorders (2, 3). Men and women from the general population had different depressive network structures. And women had larger and more intimate social networks (4). Women were more vulnerable with both no social support (5) and events occurring to others in their social network (6). They were more sensitive to the depressogenic effects of low levels of social support (4). A lower psychological everyday support was associated with feelings of worthlessness in females (7). Above may be related to higher loneliness (8), high expectations and devotion to social connection (9), more responsible toward social partners (10), low self-esteem (11), more negative life events (5), more relational events (12) and helplessness training (13) among women. Women may have worse mental health due to the lack of social connection. Furthermore, the loss of friendships had no statistically significant association with depressive symptoms among men (7).

Additionally, ‘restless sleep’ was more strongly connected to Clinical depression in the women’s network than in men’s so that women may experience this symptom but not presenting any other core depressive symptoms and clinicians may make their diagnosis only based this (14), which hints at overdiagnosis and medicalization. Clinical judgment in the diagnosis of depression may not be gender neutral and this potential gender bias may contribute to the underdiagnosis of depression in men (14). Hence, treatment for adults with depression and anxiety should take into account gender differences (7, 11) and healthcare providers should aim to provide more support that is tailored to gender. Investigating the complex pattern of interplay between depression and anxiety symptoms and pertinent physical, cognitive, and social factors and potential gender differences can help to tailor individualized interventions and provide sex-sensitive treatment.

## 3 Analysis of risk and resilience factors for depression and anxiety symptoms

It is critical to understand what the central symptoms are and what kind of risk and protective factors within network models are related to depression and anxiety symptoms, which can provide patients with the best possible care.

Perceived stress was the highest risk factor among depression and anxiety symptoms, and perceived stress influences anhedonia and social functioning (15). While mastery was the strongest resilience factor. So we can reduce the symptoms of depression or anxiety by reducing the perceived stress and enhancing the sense of mastery. On the one hand, mindfulness training provides benefits for reducing perceived stress and psychological health in adults (16). On the other hand, behaviors that are not overly protective and encourage challenge can help promote mastery, thereby reducing the risk for subsequent anxiety (17).

Psychological everyday support among males was associated only with changes in sleep pattern (7). Poor sleep quality might contribute to depressive symptoms among adults (18), trigger fatigue and worthlessness (19). Patients who suffered from severe fatigue also often suffered from symptoms of depression and/or anxiety (20, 21). In addition, participants with smoking or frequent drinker had greater association between poor sleep quality and depressive symptoms (18). Good sleep health and high levels of physical activity were both individually associated with fewer depression symptoms (22). A healthy dietary habit and regular physical activity were also potential precautions against depression (23). Physical activity’s favorable contribution to depressive symptoms was mediated partly by sleep (24). Sleep problems are highly associated with screen time symptoms. Increased screen time was associated with a small increased risk of anxiety and depression (25) and depressed individuals often tended to intensive use of social media to escape negative mood and to find relief (26). Physical activity (e.g., jogging, cycling, and swimming) might reduce addictive risk and foster well-being (26). Therefore, ensuring good sleep quality and patterns, dietary quality, physical activity, as well as avoiding tobacco or heavy drinking or caffeine and electronic screens, may have a positive effect on preventing and treating these symptoms and could be particularly beneficial for men than women in reducing overall symptom severity.

As protective factors against depression and anxiety especially for women, emotionally supportive social relationships are also of importance. It is essential to strengthen social connections for protecting against elevated levels of social anxiety and depression (27). Sustained quality social connections (QSCs) could improve mental health outcomes (28). Meanwhile, for both anxiety and depression, higher current symptomatology was associated with greater levels of self-stigma towards the illness (29). Preconceived stereotypes and personal responsibility/blame were linked to the stigma towards depression and anxiety (30). So it is significant to provide community-wide interventions aiming to increase help-seeking behavior and raise public awareness of depression and anxiety.

Considering the use of integrative interventions that combine medication, psychotherapy, healthy lifestyle, and social support are more effective to reduce the severity of symptoms of depression and anxiety in a multifaceted way (27, 31–35).

## 4 Discussion

As anticipated by Shin and colleagues, the studies of complex interrelationships between physical health, stress and adaptive cognition, and social connection with depression and anxiety symptoms as well as potential gender differences in the overall network structure have several clinical implications. There are still a number of limitations. First, in the current study modest sample size and age strata may limit the generalizability of the finding. Second, although the study included various factors encompassing physical, psychological, and social aspects, it lacked other important factors that were not included such as economic conditions, lived experience, physical condition and so on. Third, participants which used stratified probability sampling to obtain a representative sample are middle-aged Koreans living in South Korea, which remains unclear whether the research findings can be generalized to a larger population or other cultural backgrounds due to the political, economic and cultural differences. Finally, this study was based on a cross-sectional analysis and could not provide the analysis of causal associations. More studies, which include different cohorts, larger samples, longitudinal analysis and more diverse individual and environmental factors, are warranted in the future to obtain a more comprehensive and representative network structure of depression and anxiety symptoms.

In this study I see the following clear gains: it will provide evidence-based and gender-specific health-promoting strategies as well as identify the protective or harmful factors encompassing physical, psychological, and social aspects on depression and anxiety, to reduce psychopathology symptoms, to better support psychological health, and to better diagnosis and treatment, ultimately benefiting the middle-aged with depression and anxiety.

## Author contributions

YZ: Writing – original draft. CX: Writing – review & editing.
